# Case Report: Could Hennebert's Sign Be Evoked Despite Global Vestibular Impairment on Video Head Impulse Test? Considerations Upon Pathomechanisms Underlying Pressure-Induced Nystagmus due to Labyrinthine Fistula

**DOI:** 10.3389/fneur.2021.634782

**Published:** 2021-03-29

**Authors:** Andrea Castellucci, Cecilia Botti, Margherita Bettini, Ignacio Javier Fernandez, Pasquale Malara, Salvatore Martellucci, Francesco Maria Crocetta, Martina Fornaciari, Francesca Lusetti, Luigi Renna, Giovanni Bianchin, Enrico Armato, Angelo Ghidini

**Affiliations:** ^1^ENT Unit, Department of Surgery, Azienda USL—IRCCS di Reggio Emilia, Reggio Emilia, Italy; ^2^PhD Proam in Clinical and Experimental Medicine, University of Modena and Reggio Emilia, Modena, Italy; ^3^Audiology and Ear Surgery Unit, Department of Surgery, Azienda USL—IRCCS di Reggio Emilia, Reggio Emilia, Italy; ^4^Department of Otolaryngology-Head and Neck Surgery, University Hospital of Modena, University of Modena and Reggio Emilia, Modena, Italy; ^5^Audiology and Vestibology Service, Centromedico, Bellinzona, Switzerland; ^6^ENT Unit, Santa Maria Goretti Hospital, Azienda USL Latina, Latina, Italy; ^7^ENT Unit, SS Giovanni e Paolo Hospital, Venice, Italy

**Keywords:** labyrinthine fistulae, pressure-induced nystagmus, Hennebert's sign, fistula sign, video head impulse test, case report

## Abstract

We describe a case series of labyrinthine fistula, characterized by Hennebert's sign (HS) elicited by tragal compression despite global hypofunction of semicircular canals (SCs) on a video-head impulse test (vHIT), and review the relevant literature. All three patients presented with different amounts of cochleo-vestibular loss, consistent with labyrinthitis likely induced by labyrinthine fistula due to different temporal bone pathologies (squamous cell carcinoma involving the external auditory canal in one case and middle ear cholesteatoma in two cases). Despite global hypofunction on vHIT proving impaired function for each SC for high accelerations, all patients developed pressure-induced nystagmus, presumably through spared and/or recovered activity for low-velocity canal afferents. In particular, two patients with isolated horizontal SC fistula developed HS with ipsilesional horizontal nystagmus due to resulting excitatory ampullopetal endolymphatic flows within horizontal canals. Conversely, the last patient with bony erosion involving all SCs developed mainly torsional nystagmus directed contralaterally due to additional inhibitory ampullopetal flows within vertical canals. Moreover, despite impaired measurements on vHIT, we found simultaneous direction-changing positional nystagmus likely due to a buoyancy mechanism within the affected horizontal canal in a case and benign paroxysmal positional vertigo involving the dehiscent posterior canal in another case. Based on our findings, we might suggest a functional dissociation between high (impaired) and low (spared/recovered) accelerations for SCs. Therefore, it could be hypothesized that HS in labyrinthine fistula might be due to the activation of regular ampullary fibers encoding low-velocity inputs, as pressure-induced nystagmus is perfectly aligned with the planes of dehiscent SCs in accordance with Ewald's laws, despite global vestibular impairment on vHIT. Moreover, we showed how pressure-induced nystagmus could present in a rare case of labyrinthine fistulas involving all canals simultaneously. Nevertheless, definite conclusions on the genesis of pressure-induced nystagmus in our patients are prevented due to the lack of objective measurements of both low-acceleration canal responses and otolith function.

## Introduction

Pressure-induce nystagmus (PIN), also known as Hennebert's sign (HS), represents a peculiar finding indicating a third window mechanism within the inner ear. It can be elicited either by pressure changes exerted on the external auditory canal (EAC) with tragal compressions or a Politzer bulb, or increasing intracranial/middle-ear pressure through Valsalva maneuvers. It can be found in several labyrinthine disorders with normal otoscopic findings including otosyphilis (1), perilymphatic fistula (2, 3), Meniere's disease (4, 5), vestibular atelectasis (6, 7), hypermobile stapes footplate (8), semicircular canal (SC) dehiscence (9, 10), and other dehiscences between the otic capsule and surrounding structures (11, 12). Similarly, chronic inflammatory pathologies involving the middle ear may lead to labyrinthine fistula (LF) due to otic capsule erosions, accounting for pressure transmission from tympanic cavity into the inner ear (13–19). In the latter case, other classical symptoms and signs of third window syndromes including bone-conducted hyperacusis, sound-induced vertigo (Tullio phenomenon), abnormally enhanced amplitudes and reduced threshold for vestibular evoked myogenic potentials (VEMPs) may be partially or totally hidden by underlying middle ear pathologies.

The pathogenetic mechanism underlying HS is still controversial. In particular, it is unclear whether endolymphatic or perilymphatic flows are involved and which subgroup of hair cells among ampullary and/or otolith receptors represents the target sensor (4–7, 20–28).

Unlike caloric irrigations measuring horizontal SC (HSC) activity in low-acceleration ranges, the video head impulse test (vHIT) represents a recently introduced test assessing the vestibulo-ocular reflex (VOR) of each SC for high accelerations (29). Its diagnostic accuracy is based on Ewalds's laws, stating that stimulation of each canal produces eye rotations around an axis parallel to that of the canal, that ampullopetal endolymphatic flows represent excitatory stimuli for HSC while inhibitory for vertical SCs, and that stronger oculomotor responses are derived from excitatory inputs (30).

Here, we describe three patients with LF due to different temporal bone pathologies presenting with HS and positional nystagmus despite global SCs hypofunction on vHIT. We discuss the possible pathomechanisms underlying PIN and positional nystagmus and we also review the relevant literature.

## Case Descriptions

### Patient 1

**Figure 1:**
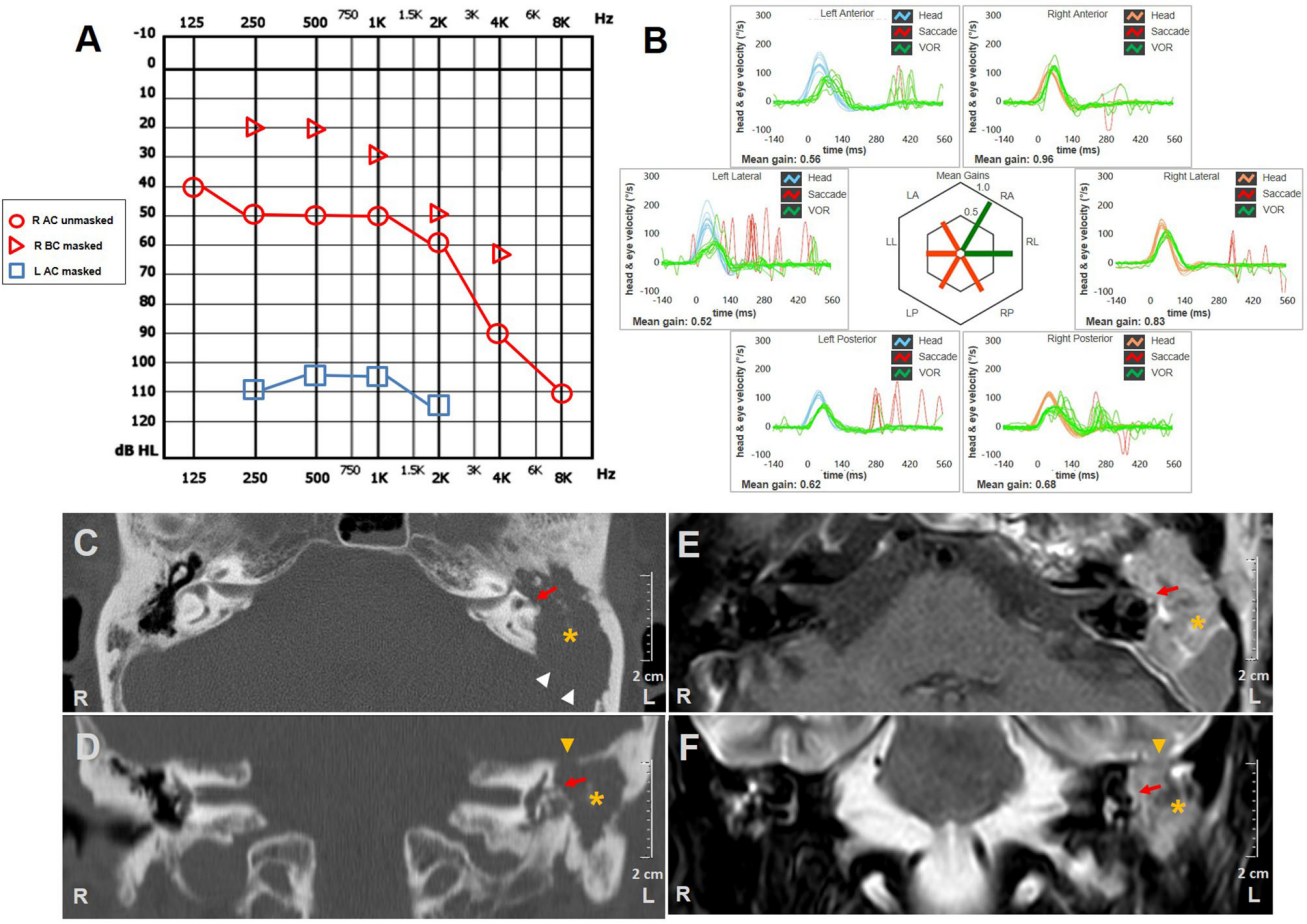
Instrumental and radiological data of case one. **(A)** Audiometry showing mixed HL on the right side and profound HL on the left. **(B)** vHIT measurements. Blue lines represent head impulses exciting left SCs, orange lines correspond to impulses for right SCs, green lines represent eye movements induced by the activation of VOR following each impulse and red lines correspond to corrective saccades. Mean value of VOR-gain (eye velocity/head velocity) is reported for each SC. The hexagonal plot in the center of the figure summarizes mean VOR-gains for each SC; normal gains are shown in green and deficient gains are in red. A global canal hypofunction on the left and a slight reduction of the VOR-gain for contralateral PSC with overt saccades could be clearly observed. Axial **(C)** and coronal **(D)** images of temporal bones CT scans completed with axial T1-weighted **(E)** and coronal T2-weighted **(F)** gadolinium-enhanced brain MRI showing soft tissue density (*yellow asterisks*) within left external/middle-ear and mastoid cavity disrupting surrounding structures. Ossicular chain is not detectable. Bony erosion areas at the posterior fossa (*white arrowheads*) and middle fossa floor (*yellow arrowheads*) with dural infiltration are highlighted. Otic capsule erosion at the left HSC is indicated with *red arrows*. AC, air conduction; BC, bone conduction; CT, computed tomography; HL, hearing loss; HSC, horizontal semicircular canal; L, left; LA, left anterior; LL, left lateral; LP, left posterior; MRI, magnetic resonance imaging; PSC, posterior semicircular canal; R, right; RA, right anterior; RL, right lateral; RP, right posterior; SC, semicircular canal; vHIT, video-head impulse test; VOR, vestibulo-ocular reflex.

An 83-year-old female presented with long-lasting left ear discharge, hearing loss (HL), and recent onset of headache. Her history was consistent with bilateral chronic otitis media (COM) and diabetes mellitus, whereas she denied oscillopsia, pressure-induced vertigo, or other vestibular symptoms. On otoscopy, her left EAC was obliterated by polypoid soft tissue. Pure tone audiometry detected ipsilateral profound HL and right-sided mixed HL (Figure 1A). Vestibular examination with video-Frenzel goggles showed neither spontaneous nor positional nystagmus. Nevertheless, left tragal compression evoked strong left-beating horizontal nystagmus that reversed on release of the positive pressure on EAC (Supplementary Video 1). Neither glottic nor nasal Valsalva maneuver resulted in detectable nystagmus or vertigo. An ICS Impulse device (Otometrics, Natus Medical Inc, Denmark) was used to measure VOR-gain values for all six SCs on the same day. Gains were considered normal if >0.8 for horizontal SCs and >0.7 for vertical canals (29). vHIT highlighted left global canal deficit and slight right-sided posterior SC (PSC) hypofunction (Figure 1B). Bedside oculomotor testing excluded signs of impaired function of central vestibular pathways. Temporal bones CT scan showed soft tissue occupying the left EAC, mastoid, and tympanic cavities with bony erosion involving ossicular chain, HSC, fallopian canal, tympanic medial wall, and tegmen tympani (Figures 1C,D). Magnetic resonance imaging (MRI) of the brain showed contrast enhanced tissue invading the intracranial compartment through superior and posteromedial walls of the temporal bone (Figures 1E,F). Histologic examination of the polypoid tissue within the left EAC was consistent with squamous cell carcinoma. Diagnosis of T4-stage disease according to the modified Pittsburgh staging system (31) was made, and the patient was addressed to palliative radiation therapy.

### Patient 2

**Figure 2:**
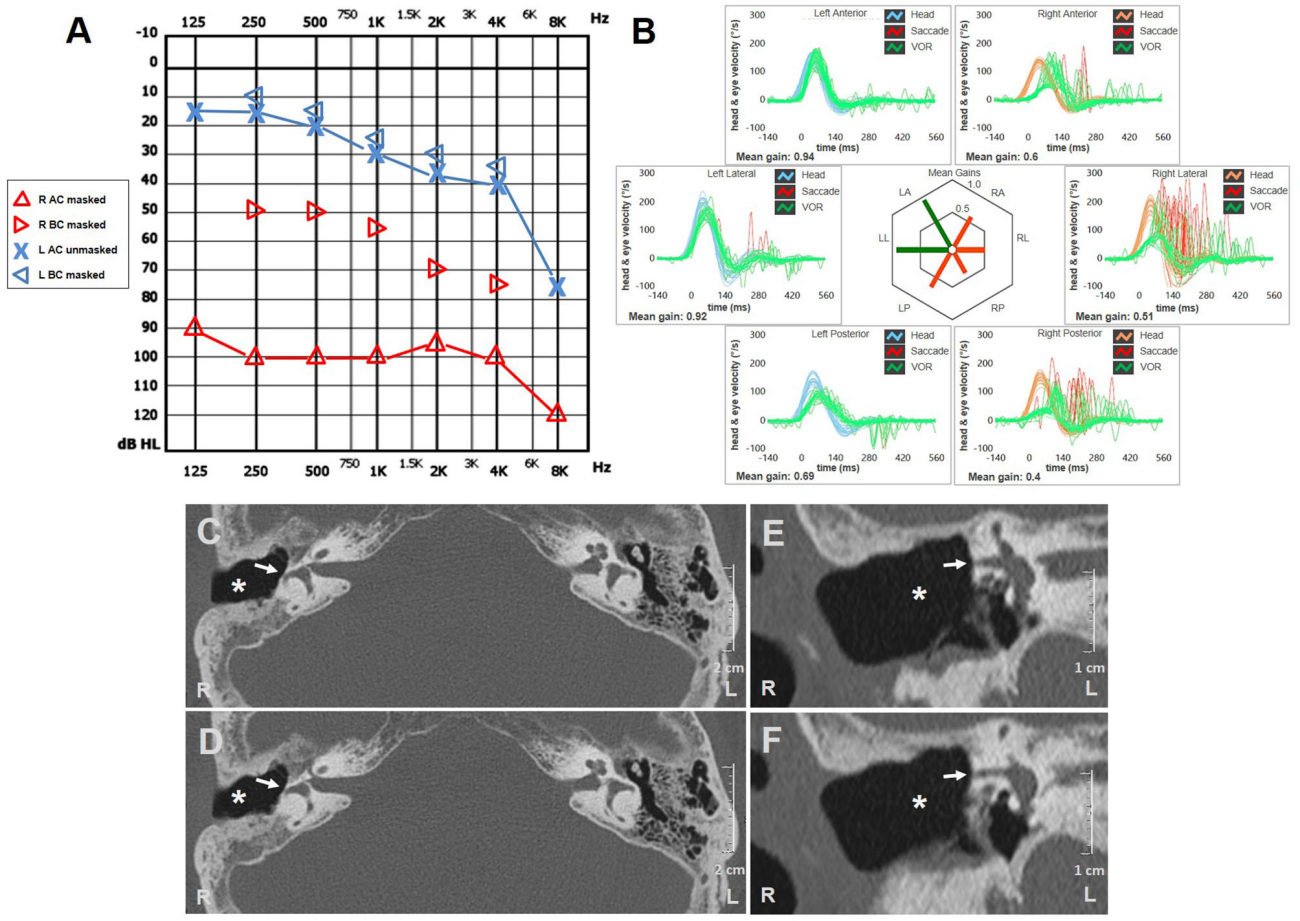
Instrumental and radiological data of case two. **(A)** Audiometric test exhibiting mixed HL on the right with predominant ABG at lower frequencies and left high-frequency sensorineural HL consistent with patient's age. **(B)** vHIT showing reduced VOR-gain values for all right SCs with both overt and covert saccades and slight hypoactive VOR for contralateral PSC without corrective saccades. Axial **(C,D)** and coronal **(E,F)** images of temporal bone CT scans detecting signs of previous CWD mastoidectomy (*white asterisks*) with HSC fistula (*white arrows*). ABG, air-bone gap; AC, air conduction; BC, bone conduction; CWD, canal wall down; CT, computed tomography; HL, hearing loss; HSC, horizontal semicircular canal; L, left; LA, left anterior; LL, left lateral; LP, left posterior; PSC, posterior semicircular canal; R, right; RA, right anterior; RL, right lateral; RP, right posterior; SC, semicircular canal; vHIT, video head impulse test; VOR, vestibulo-ocular reflex.

A 73-year-old woman referred to our center for a follow-up evaluation of right-sided canal wall down (CWD) mastoidectomy due to a COM with cholesteatoma. The surgical procedure was conducted two years earlier without simultaneous functional stage. She experienced symptoms consistent with right-sided cochleo-vestibular loss following surgery. Pressure-induced unsteadiness and dizziness represented her prominent residual vestibular symptoms. Otoscopy highlighted dry right EAC and well-preserved postoperative conditions. Her audiogram showed right-sided mixed HL with widened air-bone gap at lower frequencies and contralateral age-related sensorineural HL (Figure 2A). Although spontaneous nystagmus could not be observed with video-Frenzel goggles, mastoid vibrations elicited left-beating nystagmus. Horizontal nystagmus directed toward the affected side was evoked applying positive pressure on her right EAC, reversing on removal of the pressure. Conversely, no nystagmus could be noticed with Valsalva maneuvers. Even though she denied positional vertigo, left-beating nystagmus could be observed on supine positioning and slightly persistent geotropic direction-changing nystagmus was elicited after head rolls, with stronger amplitude on right-sided positioning (Supplementary Video 2). vHIT measurements were taken on the same day, showing VOR-gain reduction for all SCs of the right side and mildly impaired function for left PSC (Figure 2B). High-resolution CT scan detected postoperative LF involving right HSC without inflammatory recurrences (Figures 2C–F). Although we proposed revision surgery for LF closure and hearing restoration, she refused additional procedures as audio-vestibular symptoms did not prevent her from leading a normal life.

### Patient 3

**Figure 3:**
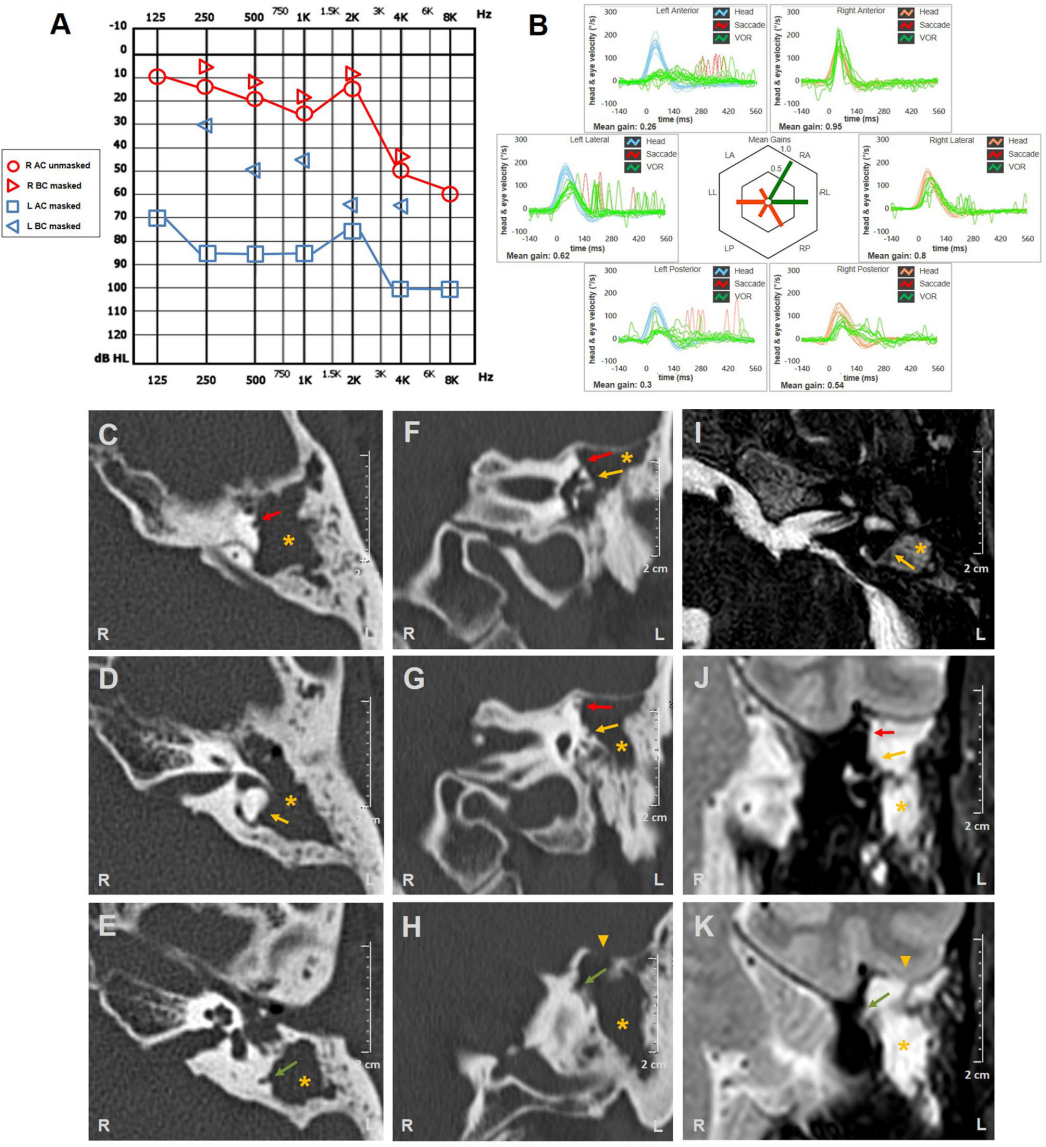
Instrumental and radiological data of case three. **(A)** Audiometric testing showing high-frequency sensorineural HL on the right side and left mixed HL with significant ABG widened for low frequencies and mild-to-moderate down-sloping sensorineural hearing impairment. **(B)** vHIT detecting reduced VOR-gain values for all left SCs with overt saccades and slight hypofunction for contralateral PSC with no corrective saccades. Axial **(C–E)** and coronal **(F–H)** temporal bones CT scans completed with axial **(I)** and coronal **(J,K)** T2-weighted gadolinium-enhanced brain MRI showing soft tissue density (*yellow asterisks*) within left middle-ear and mastoid cavity eroding ossicles and tegmen tympani (*yellow arrowheads*). Bony labyrinthine erosions of left SSC (*red arrows*), HSC (*yellow arrows*), and PSC (*green arrows*) are highlighted. ABG, air-bone gap; AC, air conduction; BC, bone conduction; CT, computed tomography; HL, hearing loss; HSC, horizontal semicircular canal; L, left; LA, left anterior; LL, left lateral; LP, left posterior; MRI, magnetic resonance imaging; PSC, posterior semicircular canal; R, right; RA, right anterior; RL, right lateral; RP, right posterior; SC, semicircular canal; SSC, superior semicircular canal; vHIT, video head impulse test; VOR, vestibulo-ocular reflex.

A 46-year-old man with a history of left ear discharge presented with newly onset HL and vertigo. His medical history was otherwise silent besides head trauma (car accident) that occurred seven months prior to the admission with residual positional vertigo. He denied oscillopsia, pressure-induced vertigo, or other vestibular symptoms. The otoscopy revealed a left thickened tympanic membrane with EAC discharge. Audiometric testing showed down-sloping sensorineural HL on the right and left-sided mixed HL with predominant conductive loss for low frequencies (Figure 3A). Right-beating spontaneous nystagmus enhanced by mastoid vibrations consistent with left acute vestibular loss could be observed on video-Frenzel examination. Nystagmus reduced in backward head bending, while increased with downbeat components in forward head tilts. Left Dix-Hallpike maneuver elicited paroxysmal up-beating nystagmus with left torsional components consistent with benign paroxysmal positional vertigo (BPPV) involving left-sided PSC, so he received Epley's repositioning procedures (Supplementary Video 3). At the following examination 2 days later, positioning tests were uneventful, whereas right-beating spontaneous nystagmus enhanced by head shakings could be still detectable. Left tragal compression induced right-torsional nystagmus, followed by stronger opposite eye movements on release of the pressure (Supplementary Video 4), whereas both nasal and glottic Valsalva maneuvers were uneventful. The patient underwent vHIT measurements on the same day, showing left global hypofunction and slightly reduced VOR-gain for the right PSC (Figure 3B). Temporal bones CT scan revealed soft tissue consistent with cholesteatoma obliterating the right-sided tympano-mastoid cavity and eroding ossicular chain, all SCs, sigmoid sinus bony wall, and tegmen tympani (Figures 3C–H). Angio-MRI of the brain ruled out venous thrombosis and meningeal involvement (Figures 3I–K). He received CWD mastoidectomy that evidenced bony erosions of each SC and permitted achievement of cholesteatoma removal. A thin matrix layer was left upon each bony defect, whereas tegmen dehiscence was repaired with bone-pate. Additional procedures for hearing restoration and LF obliteration were postponed at a later stage. Postoperative care included bed rest, intravenous broad-spectrum antibiotics, and corticosteroids tapering for additional two weeks. The patient's conditions progressively recovered and hearing threshold remained unchanged at 30 days. Spontaneous nystagmus was progressively reduced whereas vHIT findings and PIN aligning in the same axis persisted over time.

Written informed consent was obtained from each patient for the publication of this case report, including all data and images.

## Discussion

Oval and round windows represent the only sites with reduced impedance in the inner ear, whereas the remaining membranous labyrinth is entirely encased within the otic capsule. Other anatomical openings connecting the inner ear fluid spaces to the surrounding structures, such as the cochlear and vestibular aqueducts, are functionally closed to sound and pressure flows as they usually offer high impedance (11). Additional bony openings such as a labyrinthine dehiscence can result in increased inner ear compliance leading to abnormal pressure transmission into the vestibular system from surrounding compartments (2). LF represents an interruption of the otic capsule connecting perilymphatic spaces with the middle ear. It may occur in <15% of patients affected by COM with cholesteatoma (13–19) or may represent a late complication in subjects being previously submitted to CWD mastoidectomy (32, 33). Affected sites can be identified in <60% of temporal bone CT scans of patients with intraoperative findings of LF (18, 19, 34) while MRI may provide additional information about inner ear involvement (35). Whereas HSC represents the most commonly affected site, both cholesteatoma matrix and inflammatory tissue may sometimes erode other structures as PSC, superior SC (SSC), vestibule, and cochlea (13–18). As LF may also represent a pathway for toxins and pathogens invasion from the middle ear to the membranous labyrinth accounting for serous or suppurative labyrinthitis, symptoms can include variable combinations of vertigo and HL depending on LF location, on the size of bony defects, and on the extent of inner ear damage (13–19, 36). PIN represents a peculiar finding in LF, although it may show a low diagnostic sensitivity. In fact, it might be underestimated due to a possible complete functional loss of inner ear sensors/afferents or the mass-induced canal plug exerted by concurrent middle ear pathologies might prevent pressure change transmissions to the endolymphatic spaces (16, 18, 19, 28, 37). Its pathomechanism is mainly attributed to the stimulation of SC ampulla, according to the site of erosion (14, 20–23, 26–28). On the other hand, HS has been also ascribed to a possible otolith activation in other inner ear disorders, including Meniere's disease, perilymphatic fistula and vestibular atelectasis. In fact, fibrous adhesions between the stapedial footplate and the saccular membranous labyrinth (vestibulofibrosis) either due to a collapse of the membranous labyrinth or due to a saccular distension on a hydropic basis have been hypothesized (4–7, 24, 25). Nevertheless, clinical observations with VEMPs testing in a patient with endolymphatic hydrops (27) and experimental studies on LF in animal models (20) reported the onset of nystagmus after pressure changes despite saccular areflexia and after removal of the otolith membranes, respectively.

All cases herein described presented at our attention with functional loss for all SCs on vHIT besides different degrees of sensorineural HL consistent with global cochleo-vestibular damage. This condition likely represented the result of either previous or current labyrinthitis due to LF, accounting for contralesional nystagmus after mastoid vibrations detected in the second patient and for paretic spontaneous nystagmus enhanced by head shakings in the third case. Conversely, slightly reduced VOR-gains for contralesional PSCs (functionally coupled with affected SSCs) may likely result from the severe functional impairment of injured SSCs, in accordance with studies on contralesional function following vestibular neuritis and vestibular deafferentation, where an involvement of both central compensation processes and peripheral impairment of the “push-pull” mechanism have been hypothesized (38–40). On the other hand, PSC was the only hypoactive canal in the contralateral ear in all cases and the expected corrective saccades after head impulses were lacking in most cases, raising the possibility that our findings might be due to artifacts. Nevertheless, most healthy PSCs exhibited highly reduced VOR-gain values compared to ipsilateral SCs also in larger cohorts with unilateral vestibular loss (41). Moreover, the functional impairment for the PSC of the unaffected ear in the first two cases might reflect the greater effect of aging on PSC VOR-gain compared to the other SCs (42).

Unfortunately, low-acceleration VOR for HSCs could not be assessed with caloric test in the patients of our report due to concurrent external/middle ear pathologies preventing water irrigations. Nevertheless, further HSC VOR responses to different velocity and acceleration ranges could have been provided by rotational testing, but a rotatory chair was not available in our departments. Also, otolith activity could not be measured, as bone-conduction could have been the only possible way to test cervical and ocular VEMPs bypassing middle ear barriers, but it was not available in our institutions. However, given cochlear and SCs impairment, it is reasonable to assume that macular hair cells were also damaged, in accordance with studies in serous labyrinthitis (43–45).

Nevertheless, PIN could still be elicited in all subjects, likewise positional nystagmus in two cases. These apparently incongruent findings might be explained assuming a functional dissociation between ampullary hair cells encoding angular accelerations. In particular, detectable PIN despite SCs impairment on vHIT might imply a spared activity for type II hair cells and regular canal afferents encoding cupular displacements which generates nystagmus (46–48). This hypothesis is supported by studies on animal models of canal dehiscence providing evidence that sound-evoked eye movements (comparable in principle to PIN) do not only arise from sustained sound-evoked activation of phase-locking irregularly-discharging canal afferents, but also to slowly developing but sustained excitation/inhibition of regularly discharging afferents (48–50). Our assumptions are also in line with data from human temporal bone surveys showing that vestibular degeneration following serous labyrinthitis starts from type I hair-cells (44, 45). Similarly, preserved caloric responses were found in clinical studies on patients with COM (43, 51), perilymphatic fistula (52) or in cases exhibiting HS despite otolith functional loss (27). Conversely, investigations with rotatory testing have mainly documented reduced vestibular responses for the affected ear (53–55), strengthening the hypothesis of a greater impaired function of hair cells encoding for higher range frequencies. On the other hand, it may be assumed that there is a different functional outcome for damaged ampullary hair cells/afferents following acute labyrinthitis, where a selective recovery of sensors encoding for low-velocity inputs was matched by deficient high-acceleration VOR responses at long-term evaluation (38, 56). Nevertheless, a possible role of the residual function of phasic afferents in the genesis of PIN could not be ruled out a priori as all patients herein described did not present with complete canal loss on vHIT. In fact, the amount of residual canal function that is actually needed to still generate a response is not yet fully understood. Moreover, it could not be excluded that the different maximal eye velocities observed during each single canal impulse in each subject could have affected evoked nystagmus amplitudes and the interpretation of eye movements during PIN (26).

**Figure 4:**
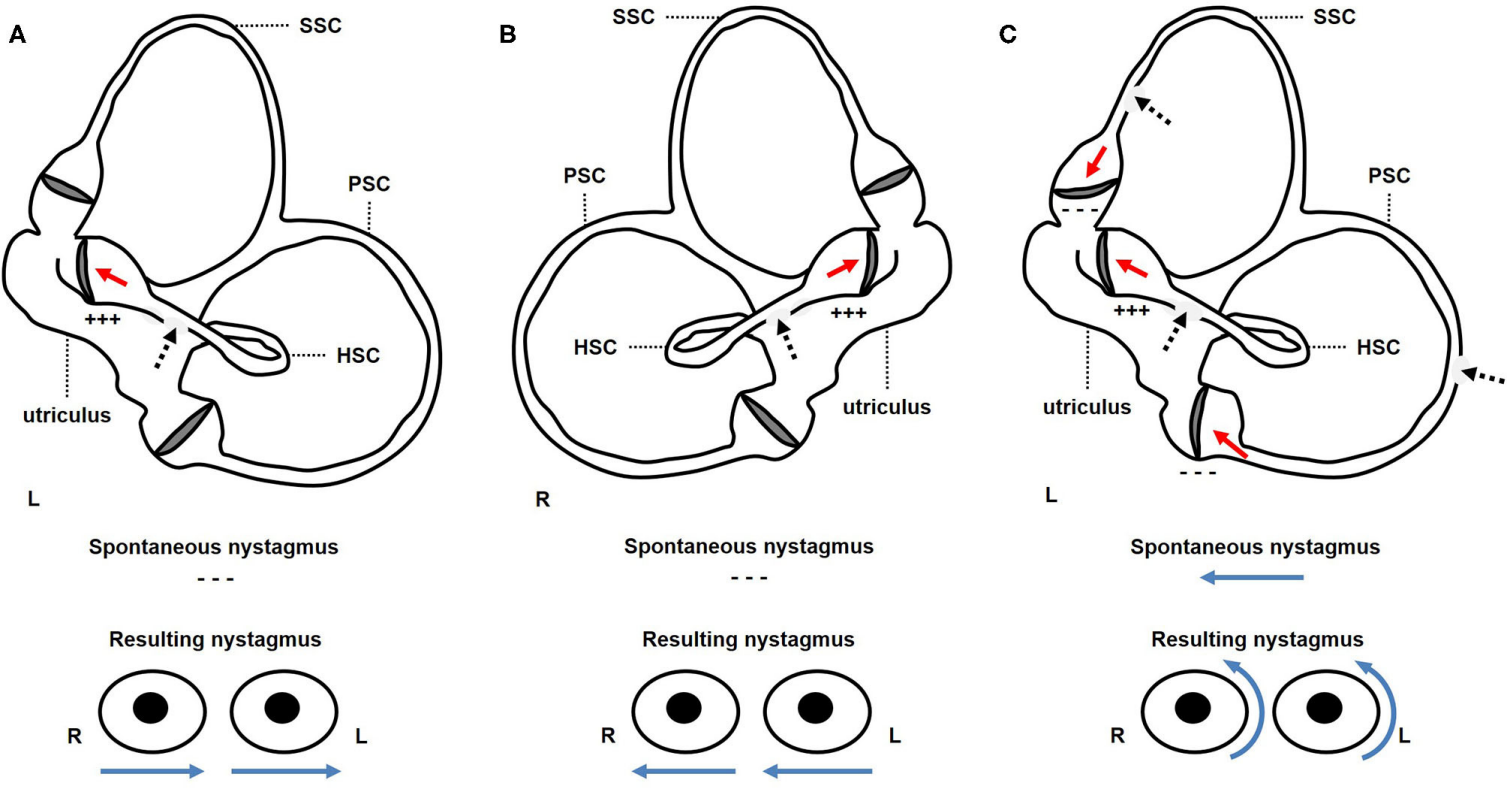
Schematic representation of the membranous labyrinth along the sagittal plane (*above*) to explain the proposed mechanism for pressure-induce eye movements (*below*) in each case. Positive pressure applied over the EAC is represented with *black dashed arrows*, resulting in endolymphatic flows with *red arrows* and direction of nystagmus fast phase with *blue arrows*. **(A)** In the first case, positive pressure applied on the left EAC resulted in ampullopetal endolymphatic flows within left dehiscent HSC, leading to transitory excitation (+ + +) of left HSC afferents. As there were no spontaneous nystagmus, resulting nystagmus was purely horizontal left-beating. **(B)** Likewise, in the second patient, right tragal compression led to ampullopetal excitatory (+ + +) endolymphatic flows within right HSC resulting in transient horizontal right-beating nystagmus. **(C)** In the third case, positive pressure exerted over the patient's left EAC generated simultaneous ampullopetal endolymphatic flows within each dehiscent canal, being excitatory (+ + +) for left HSC and inhibitory for left PSC and SSC (− − −). While horizontal left-beating components coming from left HSC activation opposed to ongoing spontaneous paretic nystagmus, vertical components generated by vertical canal inhibition (upbeat and downbeat for SSC and PSC, respectively) reciprocally canceled. Then, resulting nystagmus mainly reflected torsional rightbeating components coming from inhibition of left vertical canals afferents. EAC, external auditory canal; HSC, horizontal semicircular canal; L, left; PSC, posterior semicircular canal; R, right; SSC, superior semicircular canal.

Whereas in the first two subjects the easiest explanation for HS is represented by pressure-induced endolymphatic flows toward the ampulla of the involved HSC activating spared/recovered regular afferents (Figures 4A,B), simultaneous cupular deflection toward the utricle in all dehiscent SCs could likely account for contralesional torsional nystagmus resulting from left tragal compression in the latter case. In particular, while ipsilesional nystagmus resulting from excitatory pressure-induced input within left HSC was mitigated by underlying baseline spontaneous nystagmus beating in the opposite direction, opposed vertical components generated from simultaneous inhibiting flows within vertical SCs canceled each other. Hence, such a PIN vector (right-torsional nystagmus) could be likely derived from the sum of concurrent inhibitions of left-sided vertical SCs afferents (14, 26) (Figure 4C). In accordance with Ewald's laws, pressure removal from the affected ears elicited coplanar weaker opposite nystagmus due to inhibitory ampullofugal flows within HSC in the first two cases. Conversely, the same maneuver generated stronger ipsilesional torsional nystagmus as a result of overlapping excitatory ampullofugal inputs within the left vertical SCs in the latter case (30).

The same reasoning could be applied in the interpretation of nystagmus behavior in the last patient during head movements along the pitch plane. In this case, vertical/torsional positional nystagmus due to simultaneous left-sided PSC BPPV evoked by head bending likely superimposed underlying baseline paretic spontaneous nystagmus (57). In the same patient, paroxysmal nystagmus could be elicited despite PSC VOR-gain loss on vHIT, strengthening the assumption of spared or recovered low-velocity afferents (58). Similarly, in the second case, positional geotropic direction-changing horizontal nystagmus closely matched with the expected oculomotor findings resulting from a buoyancy mechanism likely due to penetration of toxic agents and/or inflammatory mediators into the affected HSC (59–61). On the other hand, positional nystagmus has been reported in patients with labyrinthine-intracranial fistula. In fact, it has been hypothesized that intracranial pressure variations related to sudden changes in head positions could be conveyed into the dehiscent canal and result in excitatory/inhibitory endolymphatic flows accounting for direction-changing nystagmus (62–64). Similarly, head movements have been assumed to evoke subtle mass-induced pressure changes on the membranous labyrinth at the LF area in a patient with cholesteatoma eroding the HSC who presented with geotropic positional horizontal nystagmus (65). Nevertheless, the patient of the second case herein reported should not have developed similar mechanisms, as the LF was in contact neither with intracranial spaces nor with middle ear masses. Furthermore, an outward protrusion of the membranous duct through the right HSC fistula, that should be expected to occur in right-sided head positionings due to the gravity vector, should have resulted in ampullofugal flows, and in turn to apogeotropic nystagmus, which was not the case for our patient.

Although a reverse functional dissociation pattern impairing low-velocity while sparing high-velocity canal VOR has been often observed in several vestibular diseases (66–69), other vestibular pathologies have been related to a loss of sensitivity for high-acceleration head movements while low-acceleration behavior remains intact, likewise LF herein reported (70–72). In particular, SSC dehiscence represents another condition accounting for a third window mechanism that has been demonstrated to result in ocular movements aligning with the plane of the affected canal in response to loud sounds and/or pressure changes despite selectively impaired canal function on vHIT. However, in this condition, either a plug effect exerted by middle fossa structures on membranous labyrinth or dissipation of mechanical energy through the dehiscence have been assumed as underlying factors accounting for reduced high-velocity VOR-gain for the affected canal (73, 74). Whereas this latter mechanism could hypothetically account for the global canal hypofunction on vHIT in the last case with erosion of all the three SCs, it could neither explain the impaired responses in high-velocity domain for vertical canals detected in the first two cases with isolated HSC fistula, nor could it account for the concomitant sensorineural HL.

While both cases 2 and 3 presented with a widened low-frequency air-bone gap, as expected from a third mobile window pathology (11), several symptoms and signs pertaining to the third window spectrum could not be detected in our patients. Whereas the lack of pulsatile tinnitus, own body sounds hyperacusis and vertigo induced by loud sounds could be explained by the masking effect of the underlying middle ear disorders, nystagmus induced by Valsalva maneuvers could have been missing due to the different location of LF created by middle ear disease (as in our patients) compared to SSC dehiscence at the arcuate eminence. In fact, the lack of PIN in nasal Valsalva could likely be due to the fact that pressure transmission from the nasal cavity to the middle ear through the Eustachian tube was prevented by the coexistent middle ear pathologies. On the other hand, whereas glottic Valsalva maneuver should generate nystagmus through increased intracranial pressure conveyance to the labyrinthine spaces via SSC dehiscence (9), in no case herein described did LF expose the membranous labyrinth to intracranial cavity. Nevertheless, the lack of video-oculographic recording, providing an accurate detection of subtle eye movements to pressure/sounds and slow phase velocity measures, could have prevented detecting these signs in our study.

In general, the results of our study and literature data suggest that conclusions about SCs activity in the case of vestibular impairment only based on vHIT data could be misleading, as these measurements do not reflect the whole VOR response spectrum and dissociation among afferents encoding high and low-acceleration responses could be possible. In fact, as observed in our patients, HS could be elicited despite global canal hypofunction on vHIT, allowing clinicians to combine the analysis of PIN behavior and imaging to identify the location of LF prior to middle ear surgery (14). According to the same reasoning, vestibular hypofunction on vHIT should never authorize clinicians to neglect evaluating for provoked nystagmus in patients with vestibular symptoms, as residual/spared canal activity could account for ampullary activation to endolymphatic flows despite vHIT data.

Nevertheless, as already evidenced, the main limitation of this study, preventing any definite conclusion on the genesis of PIN in our patients, is the lack of objective measurements of both low-acceleration SCs VOR and otolith function. Despite the unlikelihood of a macular contribution to vestibular responses to pressure changes in the patients herein described, we could not exclude a possible activation of otolith receptors, as it has been described how these structures could modify ongoing ocular movements or generate spontaneous horizontal nystagmus (75, 76), and how they could be functionally spared in case of labyrinthitis with concurrent third window pathologies (77). Additionally, even though the presenting instrumental picture and PIN behavior in the patient with EAC tumor overlapped vestibular findings in the other two individuals with COM, we could not exclude that the two pathologies may result in different labyrinthine lesion patterns. Even though it has been reported how squamous cell carcinoma of the EAC could result in inner ear invasion through HSC erosions (78) and how middle ear tumors could lead to both labyrinthitis and labyrinthine ischemia (79, 80), inner ear histopathology in the case of temporal bone malignancy is still mostly unknown.

## Conclusions

Although pathomechanisms underlying PIN in LF are still unclear, our case series showing that HS could be detectable even with hypoactive SCs on vHIT might offer additional insights to this aspect. Although vHIT measurements would suggest an impairment of canal function, SCs seem to represent the target sensor of HS in LF as nystagmus axis matched that of the affected canals and its characteristics strictly followed Ewald's laws. In our opinion, this apparently paradoxical finding might be possible through a functional dissociation between low- (active) and high- (impaired) velocity canal afferents. An asymmetrical damage or recovery among different subgroups of hair cells following labyrinthitis might represent the underlying process accounting for this functional behavior. Nevertheless, we strongly believe that further studies on PIN are required to substantiate the assumption that HS in LF results from the stimulation of type II hair-cells and regular afferents of dehiscent SCs.

## Data Availability Statement

The original contributions presented in the study are included in the article/Supplementary Material, further inquiries can be directed to the corresponding author/s.

## Ethics Statement

The studies involving human participants were reviewed and approved by Area Vasta Nord Emilia Romagna. The patients/participants provided their written informed consent to participate in this study. Written informed consent was obtained from the individual(s) for the publication of any potentially identifiable images or data included in this article.

## Author Contributions

AC, PM, SM, and EA: conceptualization and data interpretation. AC, CB, PM, SM, FC, MF, and FL: investigation and original draft preparation. AC, CB, MB, IJF, PM, LR, and GB: data acquisition. AC: images and artwork. GB, EA, and AG: supervision and manuscript review. All authors approved the final version of the manuscript.

## Conflict of Interest

The authors declare that the research was conducted in the absence of any commercial or financial relationships that could be construed as a potential conflict of interest.

## Supplementary Material

Supplementary Video 1 Video Frenzel findings in patient 1.

Supplementary Video 2 Video Frenzel findings in patient 2.

Supplementary Video 3 Video Frenzel findings in patient 3 (first evaluation).

Supplementary Video 4 Video Frenzel findings in patient 3 (last evaluation).

## Abbreviations

BPPV: benign paroxysmal positional vertigo
COM: chronic otitis media
CT: computed tomography
CWD: canal wall down
EAC: external auditory canal
HL: hearing loss
HSC: horizontal semicircular canal
LF: labyrinthine fistula
MRI: magnetic resonance imaging
PIN: pressure-induced nystagmus
PSC: posterior semicircular canal
SC: semicircular canal
SSC: superior semicircular canal
vHIT: video head impulse test
VOR: vestibulo-ocular reflex.
